# The Mechanisms and Application Value of Postbiotics in Caries Prevention and Management

**DOI:** 10.3290/j.ohpd.b5740317

**Published:** 2024-09-12

**Authors:** Xinchun Jiang, Mingkai Lin, Ping Xiao, Zhiyan Zhou, Yanli Zhang, Wenjuan Yan

**Affiliations:** a Student, School of Stomatology, Southern Medical University, Guangzhou, 510515, China. Study design, wrote the manuscript.; b Student, School of Stomatology, Southern Medical University, Guangzhou, 510515, China. Study design, wrote the manuscript.; c Student, Department of Stomatology, Nanfang Hospital, Southern Medical University, Guangzhou, 510515, China. Wrote the manuscript.; d Student, Department of Stomatology, Nanfang Hospital, Southern Medical University, Guangzhou, 510515, China. Wrote the manuscript.; e Professor, Stomatological Hospital, School of Stomatology, Southern Medical University, Guangzhou, 510260, China. Study conception and design, proofread the manuscript.; f Professor, Department of Stomatology, Nanfang Hospital, Southern Medical University, Guangzhou, 510515, China. Study conception and design, proofread the manuscript.; # These authors contributed equally and share first authorship.

**Keywords:** application, dental caries, microbiome, personalised treatment, postbiotics

## Abstract

Dental caries, one of the most prevalent diseases globally, affects individuals throughout their lifetimes. Recently, researchers have increasingly focused on postbiotics for caries prevention. Postbiotics, comprising inanimate microorganisms and/or their components, confer health benefits to the host. Growing evidence suggests postbiotics’ potential anticaries effects. Specifically, numerous postbiotics have demonstrated the ability to inhibit dental caries onset and progression by modulating oral flora microecology and reducing human caries susceptibility. This review elaborates on the current research regarding postbiotics’ anticaries effects, highlights some studies’ shortcomings, and innovatively proposes that postbiotics could potentially influence tooth development and salivary characteristics through epigenetic modifications. Furthermore, it anticipates postbiotics’ future application in personalised caries treatment, given their multifaceted anticaries potential.

Dental caries is a prevalent disease globally, affecting individuals of any age, gender, ethnicity, geographical location, and profession.^137^ Additionally, it is also a multifactorial disease that is primarily associated with environmental risk factors and host susceptibility, including biofilms, cariogenic diets, dental structure, and immunity and salivary characteristics of the host.^110^ Recently, there has been a heightened emphasis on caries prevention through the regulation of the host susceptibility and oral environment. The latter involves selectively inhibiting oral pathogens instead of eradicating entire microbial communities, and/or targeting the virulence factors of pathogens, aiming to preserve oral microecological balance. Consequently, researchers have initially concentrated on probiotics.^85^ Probiotics, which are live microorganisms, can enhance host health when consumed in adequate amounts.^44^ Their anticaries effects are mediated through various mechanisms, including competition, co-aggregation, antimicrobial substance production, immune system modulation, and biofilm-specific gene expression downregulation.^23,92,101,129^ Nonetheless, the use of probiotics is constrained by factors such as vitality, storage stability, tolerance, conditions of action, strain-specific mechanisms, and safety concerns, including the production of virulence factors, risk of opportunistic infections and bacteraemia in immunocompromised individuals, as well as antibiotic resistance transfer.^81,82,90,118^ Consequently, researchers are actively seeking natural therapeutic alternatives that offer greater efficacy and reduced toxicity. Considering that postbiotics, primarily derived from probiotics, exhibit significant benefits in these areas,^8,53^ research into their anticaries effects has consequently ensued. This paper will summarise and analyse in detail the mechanisms and application value of postbiotics in caries prevention and management, while forecasting promising directions for future research and potential applications in precision medicine.

## METHODOLOGY

### The Latest Concept of Postbiotics

Prior to the publication of the consensus statement by the International Scientific Association of Probiotics and Prebiotics (ISAPP) on the definition and scope of postbiotics, the term lacked a consistent definition and was known by several synonyms, including paraprobiotics, non-viable probiotics, heat-killed probiotics, bacterial lysates, and tyndallised probiotics. The most recent consensus statement by ISAPP precisely defines postbiotics as ‘preparation of inanimate microorganisms and/or their components that confers a health benefit on the host’.^105^ Specifically, postbiotics comprise intentionally inactivated microbial cells, which may or may not include metabolites (eg, secretory proteins, bacteriocins, organic acids, biosurfactants) or cellular components (eg, peptidoglycans, lipoteichoic acids, surface proteins). It should be noted that postbiotics are mixtures. Although basic purified components of microorganisms can be found in postbiotic formulations, they do not qualify as postbiotics because purified molecules should be identified using established, unambiguous chemical nomenclature. Furthermore, characterising the microorganism or microbiota, including fully annotated genome sequences, is essential before the preparation of postbiotics.

Postbiotics offer advantages over probiotics, including a longer shelf life, clearly defined safe dosage parameters, enhanced stability, and easier transportation. Studies have demonstrated that the antimicrobial effects of probiotics are partly due to the substances they produce. Furthermore, in some instances, the mechanism of action of postbiotics mirrors that of probiotics.^62^ Consequently, postbiotics may serve as a safer alternative to probiotics and have become a focal point in contemporary anticaries research.

### Specific Mechanisms of Caries Prevention and Management by Postbiotics

Postbiotics, containing various active substances, can mediate anticaries effects via multiple mechanisms. The interactions among these mechanisms can result in synergistic effects or antagonistic effects, depending on their correlation degree.

### Regulation of the Oral Microbiota Ecosystem

#### Influence on acid production and acid resistance of cariogenic bacteria

**Fig 1:**
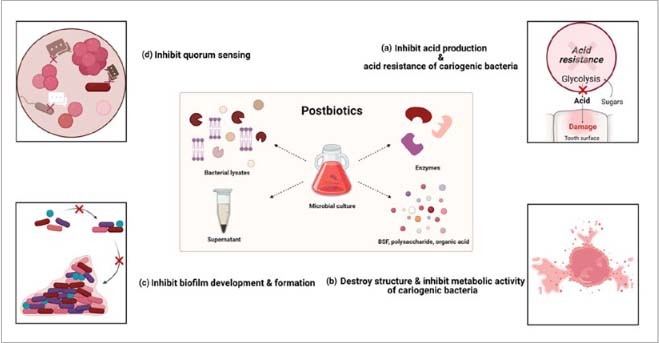
The direct effects of postbiotics in caries prevention and management. Postbiotics can directly exert an anticaries effect by regulating the oral microbiota ecosystem. This regulation includes: (a) influencing the acid production and acid resistance of cariogenic bacteria; (b) affecting structural stability and metabolic activity of cariogenic bacteria; (c) impacting biofilm development and formation; and (d) modulating quorum sensing.

It is widely recognised that acid production and acid resistance are the main virulence factors of cariogenic bacteria (Fig 1). Several postbiotics derived from probiotics have been shown to suppress the occurrence and progression of dental caries by influencing the expression of virulence genes associated with acid production and resistance in cariogenic bacteria (Table 1). For instance, postbiotics from *Lactobacillus paracasei* ET-22 (ET-22-HK and ET-22-S), have been found to significantly downregulate the expression levels of *brpA*, *LDH*, *relA*, *recA*, and *ffh* genes.^145^ Novel iminosugar compounds from *Lactobacillus paragasseri* MJM60645, the supernatant of Lactobacillus kefiranofaciens DD2, and the postbiotic mediator (PM) of *Enterobacter colacae* PS-74 significantly downregulated *brpA* gene, thereby inhibiting the acid resistance of cariogenic bacteria.^34,50,93^ Supernatants from *Lactobacillus* species are capable of affecting the expression of *atpD* and *aguD* genes, with effects varying depending on the specific *Lactobacillus* strain and the forms of *Streptococcus mutans* (*S. mutans*) (either planktonic or biofilm).^136^

**Table 1:** Effect of postbiotic-regulated genes on acid production and acid resistance in cariogenic bacteria

Gene name	Description
*brpA*	The gene encodes biofilm regulatory protein A, which is implicated in acid and oxidative stress tolerance and biofilm formation in *S. mutans*^138,139^
*LDH*	The gene encodes lactate dehydrogenase, which is a crucial enzyme in the glycolytic pathway of organisms^25^
*relA*	The gene encodes guanosine tetra (penta)-phosphate synthetase, which play a pivotal role in acid resistance in *S. mutans*^75^
*recA*	The gene encodes recombinase A, which is believed to activate DNA repair mechanisms in *S. mutans*^15^
*ffh*	The gene encodes a signal recognition particle subunit, which plays a crucial role in maintaining the composition of functional membrane proteins that contribute to acid resistance in *S. mutans*^37^
*atpD*	The gene encodes F-ATPase, which is crucial for modulating the acid adaptive response of *S. mutans*^86^
*aguD*	The gene encodes the guanidine deiminase system, which is implicated in the acid resistance of *S. mutans*^9^

Postbiotic components can also disrupt the pH balance in cariogenic bacteria by influencing enzyme activity. Specifically, palmitic, oleic, and linoleic acids markedly inhibit the F-ATPase activity of *S. mutans*. Notably, oleic and linoleic acids are more effective than NaF at inhibiting the F-ATPase activity.^38,79^

#### Influence on the structural stability and metabolic activity of cariogenic bacteria

Numerous postbiotic compounds have been shown to decrease biofilm biomass by undermining the structural integrity and metabolic functions of cariogenic bacteria. PMs derived from *L. rhamnosus* GG and *L. reuteri* were observed to significantly diminish the metabolic activity of *S. mutans*.^6^ The supernatant from *L. reuteri* AN417, a probiotic strain recently characterised, demonstrated a reduction in *S. mutans* growth rate, intracellular adenosine triphosphate (ATP) levels, cell viability, and time required for killing.^144^ The cell-free, pH-neutralised supernatants from *L. rhamnosus* Lr32 and HN001, and *L. acidophilus* LA5 and NCFM, were found to alter the expression profiles (*cdtB*, *ltxA*, and *katA*) of *Aggregatibacter actinomycetemcomitans* (Aa), resulting in a reduction of viable Aa counts, biofilm biomass, and impacted preformed Aa biofilms in a strain-specific manner.^49^ Concentrated supernatant from *Streptococcus dentisani* was found to inhibit the growth of *S. mutans*, a process believed to be mediated by bacteriocins present in the supernatant. This conclusion was drawn because the inhibitory molecules were identified as peptidic, small (<3 KDa), and capable of creating holes in the membranes of sensitive bacteria.^76^ Bacteriocins derived from *Streptococcus lactis* have demonstrated significant antimicrobial activity against Gram-positive bacteria through their interaction with the cell membranes, leading to the formation of membrane pores.^61^ The supernatants of *W. cibaria* CMU and *L. paracasei* OSU-PECh-3B are enriched with N-acetylmuramidase and hydrolase-amidase, respectively. N-acetylmuramidase is an antimicrobial protein that cleaves the 1,4-linkage between N-acetylmuramic acid and N-acetylglucosamine in the peptidoglycan found in the bacterial cell wall. Hydrolase-amidase, structurally related to other amidohydrolases such as N-acetylmuramoyl-L-alanine amidase, is involved in the degradation of peptidoglycan and hydrolysis of the amide bond between N-acetylmuramic acid and L-amino acids of the bacterial cell wall.^28,70,125^ Reuterin, secreted by *Lactobacillus reuteri*, can interfere with bacterial DNA replication by inhibiting the activity of ribonucleic acid reductase, essential for DNA synthesis.^45^ A 3-month clinical study demonstrated that bacteriocin from *Streptococcus salivarius* M18 antagonises cariogenic bacteria.^24^ L-arginine in certain postbiotics may disrupt bacterial DNA replication by upregulating the expression of the *spxB* gene (which encodes pyruvate oxidase, primarily responsible for H_2_O_2_ production^59^ in *Streptococcus gordonii*, and by reducing the interspecies competitiveness of *S. mutans*, which lacks hydrogen peroxide scavenging enzymes.^41,42^

Fatty acids, which typically comprise a significant portion of postbiotic components, disrupt the structural and metabolic activities of cariogenic bacteria and inhibit sucrose-induced demineralisation.^29^ However, it is crucial to note that certain fatty acids may exhibit pathogenicity.^7^ Consequently, it is necessary to screen for harmful components and find methods to eliminate them during postbiotic preparation. Although some postbiotics contain lactic acid, its anticaries role remains highly controversial. This controversy primarily stems from the prevailing view that lactic acid contributes to the pathogenesis of caries. However, numerous studies have highlighted the antimicrobial effects of lactic acid. Additionally, the role of lactic lactobacilli, natural residents of the oral microbiota, in cariogenesis has not been conclusively clinically demonstrated.^90^ In the authors’ opinion, controlling acidity is crucial for the anticaries effects of lactic acid and other organic acids. Specifically, postbiotics should maintain an optimal acidity level to preserve the presence and effectiveness of organic acids. Additionally, it is essential to regulate the pH to prevent it from becoming too low, avoiding enamel demineralisation and shifting the bacterial flora towards acid resistance. Moreover, effective pH values for postbiotics may vary depending on the source of the probiotics, necessitating additional experimental and clinical data to explore and substantiate.

#### Influence on biofilm development and formation

**Fig 2:**
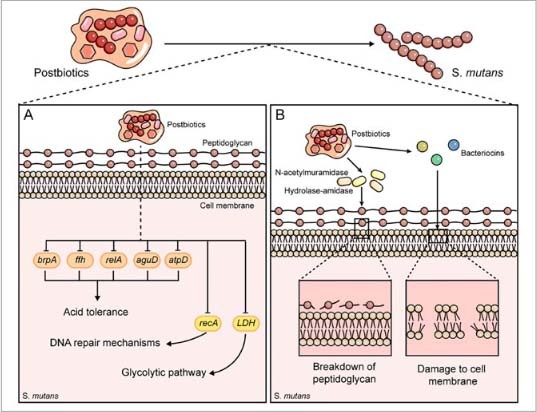
The mechanisms by which postbiotics affect acid production, acid tolerance, structural integrity and metabolic activity of *S. mutans*. (a) Postbiotics downregulate the expression of genes including *brpA*, *ffh*, *relA*, *aguD* and *atpD*, consequently reducing acid tolerance. Additionally, the downregulation of *recA* and LDH impairs DNA repair mechanisms and the glycolytic pathway, respectively. (b) In postbiotics, enzymes including N-acetylmuramidase and hydrolase-amidase degrade peptidoglycan, thereby destroying the cell wall. Simultaneously, bacteriocins disrupt the membrane by forming pores.

Biofilm is a highly dynamic, structured community of microbial cells that, once formed, acts as a refuge for bacteria, significantly enhancing their resistance to antimicrobial agents and their ability to evade the host’s immune system. In addition, increased cell densities in biofilms facilitate horizontal gene transfer, enhancing biofilm resistance or modifying the virulence spectrum.^26^ Biofilm formation is a complex process involving the formation of an initial film, bacterial adhesion to the surface and the release of extracellular polymers (EPS), bacterial colonisation, and biofilm maturation. Considering the significant role of biofilms in the development of caries, numerous studies have concentrated on the influence of postbiotics on biofilm development and formation (Fig 2).

**Lipoteichoic acid:** Lipoteichoic acid, derived from *L. plantarum*, has been shown to interfere with the catabolism of sucrose in *S. mutans*, thereby inhibiting the production of extracellular polysaccharides. During this process, lipoteichoic acid likely inhibits EPS production by reducing the activity of glucosyltransferase enzyme (GTF) and glucan-binding-protein (GBP), without affecting their expression. Additionally, the study revealed that the D-alanine components within lipoteichoic acid are crucial for its functionality. Consequently, variations in D-alanine content and repeat unit length in the molecular structure of LTA might account for the functional disparities of LTA derived from various sources. The functional mechanism of D-alanine is believed to be linked to its positive charge, as numerous studies have indicated that such a charge may encapsulate cariogenic bacteria via electrostatic interactions, or penetrate cells to bind to DNA, thereby regulating gene expression related to biofilm formation.^2^

**Polysaccharide:** A water-soluble extracellular polysaccharide, derived from *L. reuteri* BM53-1 was observed to reduce *gtfD* expression, decrease dextran viscosity, and alter dextran properties, thereby inhibiting the production of viscous β-glucans by *S. mutans*.^87^ However, *in vitro* studies indicate that *gtfB* and *gtfC* are essential for *S. mutans* cell attachment and co-aggregation, whereas *gtfD* is not necessary, suggesting that the polysaccharide may have only a minor role in colony regulation.^121^

**Biosurfactant (BSF):** Biosurfactants, complex secondary metabolites with amphiphilic structures, exhibit emulsifying properties that may aid in dispersing preformed biofilms or preventing biofilm formation.^18^ Moreover, biosurfactants modulate biofilm virulence gene expression; for example, biosurfactants from *L. acidophilus* and *L. fermentum* were shown to decrease the expression of the *gtfB* and *gtfC* genes.^121,122^ Given that the antimicrobial effects of biosurfactants significantly depend on their type and the target bacteria, selecting an appropriate study population is crucial to observe the expected effects.^133^ Additionally, while biosurfactants can be secreted into the culture medium or attached to the bacterial cell wall, most existing literature focuses on cell-bound biosurfactants, with scant data on those in the culture medium.^18,35,103,108,113,121^ In summary, biosurfactant treatment led to changes in various bacterial cell properties, including surface characteristics and gene expression. In natural systems, the complexity is significantly higher, necessitating the consideration of numerous additional factors.^32,78^

**Cell-free supernatant (CFS):** CFSs contain compounds of varying molecular weights. Extensive research shows that supernatants from diverse *Lactobacillus* species can suppress genes associated with biofilm formation, such as the *gtf* cluster (encoding glucosyltransferase), *gbp* cluster (encoding glucose binding protein), *ftf* (encoding fructosyltransferase), and *spaP* (encoding streptococcal protein antigen P).^30^ However, the extent of these effects depends on the specific *Lactobacillus* species. For example, supernatant from *L. paracasei* ET-22 suppressed the expression of the *gbp* cluster, *gtfB*, and *spaP* genes in *S. mutans*^145^; *L. kefiranofaciens* DD2 supernatant reduced the expression of *ftf*, *gbpB*, and *spaP*50; *L. plantarum* 108 supernatant inhibited and disrupted both mono-species and mixed-species biofilms of *S. mutans* and *C. albicans*. During this process, the supernatant markedly decreased the expression of *gtf* cluster in *S. mutans* single-species biofilms compared to mixed-species biofilms.^117^ Additionally, *L. plantarum* EIR/IF-1, L. curvatus EIR/DG-1, and EIR/BG-2 PMs downregulated *gtfC* expression.^90^ PMs from *L. rhamnosus* GG and *L. reuteri* significantly decreased *gtfB* expression and reduced biofilm polymeric matrix production.^6^ Similarly, PM from *Enterobacter cloacae* PS-74 markedly lowered the expression of *gtfB* and *gtfC* genes.^93^ *Enterococcus faecalis* M157 whey inhibited the expression of *gtf* cluster in a dose-dependent manner, in contrast to unfermented whey.^115^ Moreover, two novel iminosugar compounds in the CFS of *L. paragasseri* MJM60645 significantly decreased the expression of the *gtf* cluster, *gbpB*, *spaP*, and *ftf* genes.^34^ Metabolites from *L. fermentum* TcUESC38 demonstrated anti-adhesion and bactericidal effects against *S. mutans* planktonic cells, without examining associated gene expression changes.^102^ Although many studies underscore the notable anti-biofilm properties of CFSs, however, some studies suggest that supernatants from specific probiotic strains are either less effective or ineffective against multispecies biofilms.^16^ This variation could be attributed to the source of bacterial strains, inactivation methods, preparation techniques, and the composition of multispecies biofilms.

**Heat-killed probiotic:** Heat-killed probiotics are postbiotics that are prepared by inactivating microorganisms with heat treatments. Numerous studies demonstrate their significant anti-biofilm activity, with varying effects depending on the specific heat treatment.^91^ Research indicates that heat-killed *Lactobacillus* sp. interfere with *S. mutans* adhesion to saliva-coated hydroxyapatite through competition, repulsion, and substitution. This interference may occur as they occupy specific salivary receptors, essential for cariogenic bacteria adhesion.^17,123^ Additionally, heat-killed *Lactobacillus* sp. has been effective in increasing the presence of beneficial microbial strains in saliva, while simultaneously reducing the clonal growth of cariogenic strains, thus demonstrating a beneficial direction in flora regulation. However, this research did not analyse and verify the mechanism of action.^71^ Heat-killed *L. paracasei* ET-22 was observed to downregulate the expression levels of the *spaP*, *gbp* cluster, and *gtfB* genes. In this study, the authors suggested that zidovudine within the ET-22-HK signature nucleotide might serve as an active agent in inhibiting the development of *S. mutans* biofilms; however, the regulatory pathway remains unclear.^99,145^

**Organic acid:** *In vitro* studies demonstrate that l-arginine prevents *S. mutans* from adhering to salivary-coated surfaces by inhibiting the expression of gtfB and enhancing the expression of the *arcA* gene (which encodes arginine deiminase) in *S. gordonii*, thereby enriching the alkaliphilic microbial community. However, studies also reveal that although arginine impacts the production/composition of glucan, its influence on the gene expression of *gtf* cluster is minimal. This could be attributed to the timing of gene expression analysis or arginine facilitating the solubilisation of proteins encoded by *gtf* cluster via another pathway, such as modulating protein-protein interactions.^5,42,46,48^ Fatty acids, including linoleic and oleic acids, also diminish the production of extracellular polysaccharides in *S. mutans*. However, this reduction in EPS formation is not attributed to the inhibition of GTF activity but rather to the suppression of *S. mutans* biofilm cellular F-ATPase activity.^94^ This effect arises because the enzymes secreted by bacterial cells are typically linked to ΔpH across the cell membrane, and linoleic and oleic acids may disrupt biofilm cell F-ATPase activity, altering ΔpH and subsequently inhibiting GTF secretion.^79^ Additionally, some studies indicate that fatty acids may prevent bacterial adhesion to enamel by encapsulating individual bacteria in micelles.^29^

Peptide nisin, an antibiotic peptide, is produced by *Lactococcus lactis.*^19^ Experimental evidence demonstrates that it inhibits the synthesis of glucan biofilm by *S. mutans*.^142^ Cyclo(L-leucyl-L-prolyl), secreted by *Bacillus amyloliquefaciens*, effectively inhibits bacterial adhesion among bacteria and to hosts. This effect is achieved through the reduction of cell surface hydrophobicity and the downregulation of *gtfC* and *gbpB* genes.^33^ A clinical study has found that bacteriocin from *S. salivarius* M18 antagonise cariogenic bacteria. Furthermore, the dextransucrase and urease it produces facilitate glucan decomposition (which assists in plaque dissolution) and urea hydrolysis (which elevates saliva pH), respectively, thereby preventing the development of cariogenic biofilms.^24^

#### Influence on quorum sensing

Quorum sensing (QS) is a bacterial communication mechanism that regulates several processes including bacterial motility, antibiotic biosynthesis, biofilm formation, plasmid coupling, and the production of virulence factors. This mechanism enables bacteria to sense and adapt to environmental changes by modifying gene expression, which offers protection from environmental challenges, enhances defence against host immune systems, facilitates competition with other bacteria, and supports physiological activities related to virulence.^68^ The mechanism of QS operates on the principle of production, detection, and response to extracellular signalling molecules, known as autoinducers (AIs). Each bacterial species produces and responds to specific autoinducers. As an illustration, Gram-negative bacteria utilise acylated homoserine lactones (AHLs), while Gram-positive bacteria employ autoinducing peptides (AIPs) as specific autoinducers.^95^ Furthermore, AI-2, known as a ‘universal’ signal produced through LuxS-mediated methyl metabolism, is not species-specific, thereby facilitating interspecies communication and often playing a significant role in controlling virulence.^140^ The Com system, which includes ComCDE and ComRSX, serves as the predominant community-sensing mechanism for intraspecies communication in *S. mutans*.^40,111^ Decreased gene expression in this system compromises *S. mutans* communication, resulting in diminished acid tolerance, acid synthesis, genetic transformation, bacteriocin production, and biofilm formation capabilities.^63,66,67,120^ The VicRKX system regulates oxidative stress tolerance in cariogenic bacteria, regulating genes associated with virulence, including *gtf* cluster, *ftf*, and *gbpB*. This regulatory process influences *S. mutans* growth, sucrose-dependent adherence, biofilm formation, and genetic competence.^50,60,111^ Disruption of the QS systems hampers bacterial communication, diminishing virulence inhibition among bacteria, thus impairing their survival and reproduction.^100^

Recent studies have increasingly demonstrated that postbiotics are capable of reducing bacterial populations within biofilms and diminishing the production of virulence factors by inhibiting QS. For instance, lactic acid and phenyl lactic acid, produced by probiotics, have been found to inhibit homoserine lactone (HSL) production and the ComDE system, respectively.^112,145^ PMs from three probiotic strains have been observed to suppress the expression of *comA* and *comX* genes in *S. mutans* at non-lethal concentrations.^90^ Biosurfactants isolated from *Pediococcus acidilactici* and *Lactobacillus plantarum* have been shown to significantly reduce Autoinducer-2 (AI-2) synthesis in a dose-dependent manner, thereby interfering with both intraspecies and interspecies communication.^143^ Supernatants from *Enterobacter cloacae* PS-74 and *L. kefiranofaciens* DD2 were found to decrease the expression of QS signal transduction-regulated genes, including *comDE* and *vicR*, in *S. mutans*.^50,93^ Similarly, patent records indicate that the l-leucyl-l-prolyl molecular ring from *Bacillus amyloliquefaciens* produces an analogous effect.^33^ Furthermore, another study revealed variations in the effects of supernatants from four *Lactobacillus species* on the expression of *comCD* and *vicKR* genes in both planktonic and biofilm forms of *S. mutans*.^136^

The ability of postbiotics to modulate cariogenic bacterial group sensing holds significance because inhibiting group sensing represents a potential strategy against drug-resistant pathogens, yet the development and study of such drugs are currently limited. Furthermore, the accessibility and affordability of postbiotics, derived from a wide range of sources, herald a promising new phase in combating drug resistance.^4^

### Modulation of Human Susceptibility to Dental Caries

#### Modulation of oral immunity

**Fig 3:**
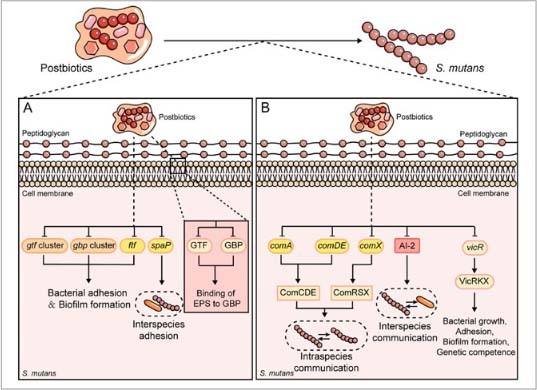
Mechanisms by which postbiotics influence bacterial adhesion, biofilm formation and quorum sensing in *S. mutans*. (a) Postbiotics downregulate the expression of genes including *gtf* cluster, *gbp* cluster and *ftf*, thereby inhibiting bacterial adhesion and biofilm formation. Moreover, the downregulation of *spaP* inhibits interspecies adhesion. Postbiotics also reduce the activities of GTF and GBP, which inhibits the binding of EPS to GBP. (b) Postbiotics inhibit the expression of comA, comDE and comX, thereby interfering with the ComCDE and ComRSX systems. This interference impedes the regulation of target gene expression and ultimately blocks intraspecies communication. Postbiotics also suppress AI-2, thereby blocking interspecies communication. Furthermore, postbiotics inhibit the expression of the* vicR* gene expression, thereby interfering with the VicRKX system. This interference affects the growth, adhesion, biofilm formation, and genetic competence of *S. mutans*.

**Fig 4:**
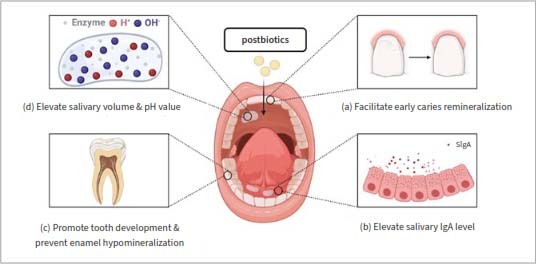
The indirect effects of postbiotics in caries prevention and management. Postbiotics can indirectly achieve an anticaries effect by modulating human susceptibility to dental caries. This modulation includes: (a) modulating oral immunity; (b) influencing the quality and quantity of saliva; (c) influencing tooth development and calcification processes; (d) influencing tooth demineralisation and remineralisation.

During the formation and development of cariogenic biofilm, the body fights infection and suppresses the growth, multiplication, and virulence of cariogenic bacteria through innate and adaptive immune responses (Figs 3 and 4). However, if the body’s immunity weakens due to internal or external factors and fails to sufficiently restrict the invasion of cariogenic bacteria, the risk of acute and rampant caries increases. Consequently, bolstering the immune response in immunocompromised individuals can help reduce their caries incidence.

Postbiotics modulate oral immunity through the promotion of oral immune factor production and secretion. UV-inactivated *Lactobacillus rhamnosus* 1505, acting as an immune adjuvant akin to bacterium-like particles (BLP), potentially enhances the production of specific systemic SIgA. This effect is achieved by stimulating human dendritic cells, differentiated from peripheral blood mononuclear cells, to secrete IL-6 and IL-10.^22,106,107^ A clinical trial demonstrated that oral administration of heat-killed probiotic tablets significantly elevated salivary IgA levels, likely through the upregulation of IL-10 and TGF-Beta.^73^

While postbiotics may reduce bacterial colonisation in the oral cavity through immune activation, they could also result in excessive immunisation. Nonetheless, evidence indicates that peptidoglycan from probiotics or commensal bacteria can trigger immunostimulatory factor production without causing harmful inflammation.^107^ This phenomenon could be attributed to the dynamic interplay among various components or the possibility that increasing a component’s dosage enhances its immunostimulatory effect; concurrently, there is an elevation in anti-inflammatory factor production.^80,119^ Nevertheless, it cannot be guaranteed that postbiotics will prevent harmful inflammation, as they comprise various substances with unverified effectiveness in mitigating excessive inflammation or boosting immune response adequately. Consequently, it is imperative for researchers to meticulously analyse the dose-response relationship of components in their studies.

#### Effects on the quality and quantity of saliva

A clinical trial demonstrated that urease, produced by *S. salivarius* M18, catalyses the hydrolysis of urea, thereby increasing saliva pH.^24^ Citrulline and arginine, constituents of specific postbiotics, mediate the PPAD-citrullination pathway^132,145^ and the arginine deiminase system (ADS) reaction,^7,46^ respectively. Arginine, notably, neutralises glycolysis acids through ADS activity, thus maintaining biofilm pH balance, and additionally interferes with EPS matrix formation, increasing in situ pH levels at the biofilm-sHA interface.^36,42,141^ Furthermore, postbiotics potentially regulate genes associated with the synthesis and secretion in salivary glands. Research suggests that postbiotics may regulate epigenetic methylation modifications,^20^ with methylation activation being beneficial for the suppression of Sjögren’s syndrome,^58^ potentially involving mechanisms linked to the activation of the IFN pathway.^64^ The CFTR (cystic fibrosis transmembrane conductance regulator) plays a crucial role in the exocrine secretion in salivary glands, with gene methylation and demethylation potentially regulating the *CFTR* gene in a tissue-specific manner at various stages of human development, influencing the development and function of salivary glands.^114^

#### Effects on tooth development and calcification processes

Epigenetic mechanisms significantly influence genes associated with the mineralisation and development of teeth (Table 2). For instance, variations in DNA methylation can lead to notable disparities in tooth number among monozygotic twins with identical genotypes.^126–128^ DNA methylation, a fundamental epigenetic mechanism, plays a crucial role in tooth development. Research has shown that variations in DNA methylation across several genes, such as *Pbx1b*, *ACAT2*, and *LTBP3*, are closely linked to tooth development and mineralisation.^84^ A study investigating the links between sporadic non-syndromic anodontia, hypodontia, and epigenetics revealed that changes in the methylation status of certain genes, including *NFκBIB*, *PRKCD*, *HEY1*, *BID*, and *CACNA1A*, can affect three signalling pathways – MARK, Notch, and Wnt/Ca2+ – involved in tooth development.^134^ However, there are many genes associated with non-syndromic hypodontia that have been found to be independent of methylation levels, such as EDA, PAX, MSX and AXN.^134^ Although unconfirmed, it’s hypothesised that these genes could be affected by other epigenetic mechanisms. Another study found an association between the methylation status of genes involved in tooth development and insufficient enamel mineralisation, but failed to establish a clear causal link.^84^

**Table 2:** The relationship of the aforementioned genes to tooth development and calcification

Gene name	Description
*ACAT2*	The gene encodes acetyl-CoA acetyltransferase 2 and may be associated with erosive tooth wear^3^
*LTBP3*	The gene is associated with human oligodontia and amelogenesis imperfecta^47,88^
*Pbx1b*	The gene is expressed in the dental lamina during the early stages of odontogenesis^109^
*NF* *κ* *BIB*	The gene encodes an inhibitor of NF*κ*B expression that is associated with tooth number, shape, and eruption^27,89^
*PRKCD*	The gene encodes multifunctional enzymes that are associated with ameloblast differentiation and BMP-4-induced osteoblastic differentiation^96,131^
*HEY1*	The gene is associated not only with the differentiation of tooth-forming cells, calcification of tooth hard tissues, apical morphology and root generation, but also with the induced differentiation of pulp stem cells into dentin-forming cells after tooth eruption^14,21^
*BID*	The gene encodes apoptosis-related proteins^77^
*CACNA1A*	The gene is associated with dental epithelial stem cell activity, proliferation, differentiation and enamel formation^39,51,116,146^

It has been suggested by researchers that elucidating the local triggers of differential methylation in genes associated with tooth development and mineralisation could aid in the early detection of enamel hypomineralisation. Changes in these triggers signify significant preventive possibilities.^84^ Recent research indicates that microbial metabolites, such as short-chain fatty acids, can influence epigenetic modifications, altering cell transcription and resulting in host genome reprogramming.^10,20,52,97^ However, studies in this area are limited and primarily focus on obesity and immunity. Considering the extensive impact of microbial metabolites on systemic DNA methylation,^134^ postbiotics hold potential in regulating enamel development and mineralisation. However, research in this domain is still in its early stages, limiting the scope of definitive conclusions. Given that environmental impact on genetics begins as early as the embryonic or intrauterine stage,^65^ early prevention of caries, through epigenetic regulation of gene expression via diet or medication, is paramount. Therefore, the prospects for research on postbiotics in this field are promising.

#### Effects on tooth demineralisation and remineralisation

In the situation of dental caries, the dental pulp will express a variety of cytokines to promote the odontogenic differentiation of dental pulp derived stem cells (DPSCs) and the development of restorative dentin. Some researchers discovered that probiotic yoghurt extracts can reduce enamel demineralisation in experimental settings.^130^ However, this study merely documented the phenomenon without explaining the underlying reasons. Bacterial lysates from *L. plantarum* and *L. rhamnosus* were reported to reduce MAPK phosphorylation and NF-κB activation, therefore improving the ability for odontogenic differentiation in DPSCs.^43,55^ A clinical investigation found that toothpaste containing 1.5% l-arginine and fluoride improved the demineralisation/remineralisation equilibrium and outperformed fluoride alone.^124^ Similarly, an experiment demonstrated that both arginine alone and in combination with NaF enhanced tooth remineralisation. The researchers believe the mechanism involved may be that Arg uptake by enamel can nucleate sub-surface crystal mass.^11,12^ It has been found that LPS significantly promotes the differentiation of DPSCs toward dentin cells in a dose-dependent manner, which involves the modulation of the biological behaviour of DPSCs by LPS-induced IFN-γ. Notably, lower concentrations of IFN-γ foster DPSC proliferation and migration to the damaged site while concurrently suppressing odontogenic differentiation; in contrast, higher concentrations of IFN-γ expedite odontogenic differentiation, which involves inhibiting the NF-κB and MAPK signalling pathways.^43^ However, research has demonstrated that the activation of NF-κB signalling can facilitate odontogenic differentiation of DPSCs in inflamed sites.^83,98,135^ As a result, the processes underpinning tooth remineralisation are highly complex, prompting further research into the involvement of postbiotics in this setting.

Table 3 presents the outcomes of *in vivo* and *in vitro* studies undertaken to examine the impact of postbiotics and their components on cariogenic factors.

**Table 3:** Studies on the anticaries effects of postbiotics

Postbiotics or its components	Microorganisms	Effects	References
Cell-free supernatant (CFS)	*Lactobacillus fermentum* 20.4, *Lactobacillus paracasei* 11.6 *Lactobacillus paracasei* 20.3 *Lactobacillus paracasei* 25.4	The activity of *S. mutans* was inhibited, and biofilm formation was significantly reduced.	104
Heat-killed probiotic and cell-free supernatant	*Lactobacillus rhamnosus* ATCC 53103 and* Lactobacillus paracasei* B21060	The heat-killed probiotics inhibit *S. mutans* biofilm formation by competition and displacement, with effects comparable to those of live probiotics. The CFCSs from two *Lactobacillus* strains, especially the undiluted *Lactobacillus paracasei* B21060, reduced *S. mutans* and *S. oralis* biofilm formation, which was associated with the production and release of antimicrobial compounds (eg, hydrogen peroxide, bacteriocins, and biosurfactants).	17
Spent culture suspension (SCS)	*Lactobacillus pentosus* 13-1, 13-4 and* Lactobacillus crispatus* BCRC 14618	The SCSs exhibited significant antimicrobial activity, where the compound synthesised by *Lactobacillus pentosus* 13-4 with potential antimicrobial properties could be lipophilic proteins.	72
Cell-free supernatant	*Lactobacillus paragasseri* MJM60645	The novel iminosugar compounds contained in the supernatant strongly downregulated the expression levels of genes associated with biofilm formation, including *gtfB, gtfC, gtfD, gbpB, brpA, spaP, ftf,* and* smu1,* without affecting the expression of *comDE* or* relA*.	34
Postbiotic mediator (PM)	*Lactiplantibacillus plantarum* EIR/IF-1,* Lactiplantibacillus curvatus* EIR/DG-1, and *Lactiplantibacillus curvatus* EIR/BG-2	All PMs decreased cell viability and biofilm formation, with the strongest effect (pH-dependent) in PMs of *Lactobacillus plantarum* EIR/IF-1. Sub-MIC values of PMs downregulated the expression of *gtfC, comA*, and* comX*, and inhibited the production of the QS machinery and virulence factors, potentially attributed to organic acids, fatty acids, and vitamins.	90
Cell-free supernatant	*Lactobacillus kefiranofaciens* DD2	Growth and biofilm formation of *S. mutans* was inhibited by downregulation of the expression of *ftf, brpA, comDE, vicR, gbpB*, and* spaP* genes, which may be related to organic acids, novel extracellular polysaccharides.	50
Bacterial lysates (BL)	*Lactobacillus plantarum* and* Lactobacillus rhamnosus GG*	MAPK and NF-κB signalling pathways were inhibited and extracellular polysaccharide synthesis-related genes (*gtf* cluster) were downregulated.	55
Chloroform extract of cell-free culture supernatant	*Bacillus amyloliquefaciens* (MMS-50)	The glycolytic activity of *S. mutans* was inhibited by reducing cell surface hydrophobicity. Biofilm formation, glucan synthesis, acid production, c, and community sensing were inhibited by suppressing *vicR, comDE, gtfC*, and* gbpB* gene expression.	33
Cell-free supernatant	*Lactobacillus casei Shirota, Lactobacillus casei* LC01, *Lactobacillus plantarum* ST-III, *Lactobacillus paracasei* Lpc-37, and *Lactobacillus rhamnosus* HN001	Untreated supernatants inhibited *S. mutans* growth of *S. mutans* and biofilm formation, whereas treated supernatants inhibited the growth of *S. mutans* and biofilm formation only in the case of *L. casei Shirota* and *L. rhamnosus* HN001.	74
Biosurfactant	*Lactobacillus reuteri* DSM 17938, *Lactobacillus acidophilus* DDS-1, *Lactobacillus rhamnosus* ATCC 53103, and *Lactobacillus paracasei* B21060	Adhesion and biofilm formation on titanium surfaces of *S.mutans* and *S. oralis* were significantly inhibited in a dose-dependent manner.	18
Nisin	*L. lactis subsp. lactis* ATCC 11454	Insoluble glucan biofilm synthesis was completely inhibited in *S. mutans.*	142
Spent culture suspension	*Lactobacillus casei* (ATCC 393), *Lactobacillus reuteri* (ATCC 23272), *Lactobacillus plantarum* (ATCC 14917) or *Lactobacillus salivarius* (ATCC 11741)	Expression of genes related to exopolysaccharide production, acid tolerance, and quorum sensing *(atpD, aguD, gtfBCD, sacB, vicKR*, and* comCD*) was downregulated, which is associated with organic acids, peroxides, and bacteriocins.	136
Fatty acid	*Arthrographis kalrae*	MS acid production was mitigated and water-insoluble exopolysaccharide production and biofilm formation were inhibited.	1
Postbiotic mediator	*Enterobacter colacae* PS-74	Expression of QS signalling, glucan metabolism, and biofilm-regulated genes *(*eg, *gtfB, gtfC, comDE, vicR, brpA*) was downregulated in the *S. mutans*.	93
Cell-free supernatant	*Lactococcus lactis Lactobacillus paracasei*	The N-acetylmuramidase from *Lactococcus lactis* supernatant hydrolyses peptidoglycan and degrades bacterial cell wall. The hydrolase-amidase from *Lactobacillus paracasei* supernatant degrades peptidoglycan and hydrolyses the amide bond between N-acetylcytidylic acid and L-amino acid in the bacterial cell wall.	28
Biosurfactant	*Lactobacillus fermentum*	The gene expression of *gtfB/C* was downregulated, and MS attachment and biofilm formation were inhibited.	122
Lipoteichoic acid	*Lactobacillus plantarum*	The formation of multispecies biofilms on dentin slices was inhibited in a dose-dependent manner. The effect of intracanal medication in removing formed multispecies biofilms was enhanced.	54
Postbiotic mediator	*Lactobacillus rhamnosus* GG (LGG) and *Lactobacillus reuteri* (LR)	Metabolic activity, *gtfB* gene expression, and biofilm formation were inhibited in *S. mutans.*	6
Postbiotic mediator	*Lactiplantibacillus plantarum* EIR/IF-1,* Lactiplantibacillus curvatus* EIR/DG-1,* and Lactiplantibacillus curvatus* EIR/BG-2	The QS and virulence of cariogenic bacteria were inhibited by the downregulation of the expression of *gtfC, comA* and* comX.*	90
Lactic acid	*Pediococcus acidilactici* M7	Inhibitory effects on QS signalling molecules and certain QS-dependent virulence factors in Gram-negative bacteria.	56
Biosurfactant	*Pediococcus acidilactici and Lactobacillus plantarum*	AI-2 synthesis was inhibited in a dose-dependent manner.	143
Biosurfactant	*Lactobacillus acidophilus DSM 20079*	*S. mutans* biofilm formation was inhibited and *gtfB/C* expression was downregulated.	121
Lipoteichoic acid	*Lactobacillus plantarum* KCTC10887BP	The activity or function of GTF and GBP is inhibited.	2
Cell-free supernatant	*Lactobacillus fermentum* TcUESC01	*S. mutans* was killed and its ability to adhere was inhibited.	102
Cell-free supernatant	*Lactobacillus reuteri* AN417	Growth rates, intracellular ATP levels, cell viability, time-to-kill and biofilm integrity were significantly inhibited in *S. mutans.*	144
Cell-free supernatant	*Lactobacillus plantarum* 108	*S. mutans* and *C. albicans* single and hybrid biofilm formation was inhibited, and *gtfBCD* gene expression was downregulated.	117
Whey	*Enterococcus faecalis* M157	MAPK phosphorylation and NF-κB activation were inhibited. Secretion of IL-1β and IL-8 was inhibited. Expression of the *gtfBCD* genes was downregulated in the *S. mutans*.	115
Cell-free supernatant	*Streptococcus dentisani* 7746 and 7747	MS growth was inhibited, which is associated with bacteriocins.	76
Cell-free supernatant	* Lactobacillus rhamnosus* Lr32, *Lactobacillus rhamnosus* HN001,* Lactobacillus acidophilus* LA5, and* Lactobacillus acidophilus* NCFM	The CFSs altered expression profile of Aa, reduced Aa viable counts and biofilms biomass and influenced Aa preformed biofilms in a strain-specific fashion.	49
Bacteriocin, dextranase and urease	*Streptococcus salivarius* M18	Bacteriocins effectively antagonised acidogenic dental plaque inhabitants. Dextranase and urease counteract plaque formation and saliva acidity, respectively.	24
Heat-killed probiotic and secretion	*Lactobacillus paracasei* ET-22	The growth of *S. mutans* biofilm growth, the synthesis of both water-soluble polysaccharide and water-insoluble polysaccharide, and the expression levels of virulence genes (*brpA, LDH, Rela, recA, ffh, spaP, gbpABC, gtfB*) were inhibited.	145
Polysaccharide	*Lactobacillus reuteri* BM53-1	The *gtfD* expression was downregulated.	87
Heat-killed probiotic	*Lactobacillus salivarius subsp. salicinius* AP-32 and* Lactobacillus paracasei* ET-66	Salivary IgA levels were significantly elevated, probably due to elevated levels of IL-10 and TGF-Beta.	73

## RESULTS

### Pathways to Enhancement, Improvement and Application

#### Current status of research

Numerous *in vitro* studies have investigated the impact of postbiotics on factors that contribute to dental caries; however, some of them only report results such as biofilm formation inhibition and biomass reduction, without delving into the effective components and underlying molecular mechanisms. Meanwhile, *in vivo* and clinical trials are less common, and many of these studies have experimental design flaws such as a lack of blinding, the absence of a placebo group, control groups made up of untreated subjects, and small sample sizes. Furthermore, research focusing on molecular pathways predominantly examines the microecology of oral flora, with only a minority addressing human caries susceptibility. Additionally, although focus has been placed on various gene expression changes and regulatory pathways of oral microbiota, numerous pathways and related genes remain largely unexplored or unvalidated.^13,31,75^

#### Future research directions

Given the current state of research, there is still a long way to go in anticaries research for postbiotics, where it is necessary to use additional experimentation to fill theoretical gaps. This includes determining effective concentrations, dosages, long- and short-term intake, modes of administration, and ensuring safety. Several factors justify the aforementioned perspectives. First, unlike probiotics, postbiotics do not colonise and grow, and their effects will cease once supplementation is stopped. Second, these substances could induce side effects like excessive inflammatory responses if their safe concentrations are not established. Third, if postbiotics derived from a certain bacterial strain are proven to be beneficial, further research is needed to explore to what extent various processing and application methods can achieve or surpass the expected effects. Fourth, most of the studied postbiotics are made by collecting cell-free culture supernatants of probiotics or direct heat treatment, electromagnetic radiation, and so on. In this process, not only the beneficial components of postbiotics are collected, but harmful substances are also easily collected, such as aromatic amino acids, sulphur-containing amino acids and hybrid polyketide nonribosomal peptides.^7^ This necessitates further research for resolution and attention. Fifth, for postbiotic components that have previously been shown to prevent cavities, the impact on other diseases must be examined. For example, while arginine metabolism can reduce sucrose metabolism in oral microbes, it may negatively impact the respiratory tract because the metabolic product of arginine, ornithine, can decarboxylate to form polyamines, thereby supporting their survival.^13^ Sixth, some challenges remain regarding whether postbiotics are as durable as probiotics.^45^ In the search for postbiotics with anticaries effects, researchers must be aware that the modulation of cariogenic factors by postbiotics derived from various microorganisms is strain specific, and that the effects are also affected by the strain mix, culture environment, and processing methods. Future studies should investigate additional probiotics or probiotic communities that produce anticavity postbiotics and find their optimal methods of inactivation and processing, among which the regulation of pH value is particularly in need of attention.^136^ Many studies have found that neutralising postbiotics reduces their antimicrobial activity, possibly because the function of organic acids is inhibited.^90^ But in view of the phenomenon of acid dissolution, the use of organic acids to fight caries is still somewhat controversial. However, the only way to significantly cause demineralisation is to lower the acidity of the enamel to a certain point, and it’s notable that organic acids can weaken the activity of cariogenic bacteria and prevent them from producing acid, which generally prevents further pH decline. Moreover, even though lactic acid bacteria are capable of producing acid, their cariogenic characteristics have not yet been established by clinical evidence. Therefore, creating mildly acidic postbiotics is meaningful as it prevents both the acidification and demineralisation of teeth, as well as the compromised efficacy of organic acids. However, the pH value must be carefully explored because the ideal pH may change amongst postbiotics source, and the final product must be supported by clear clinical outcomes.^90^ Although there are numerous *in vitro* trials indicating the beneficial anticaries effects of postbiotics, it is known that these effects are dependent on the duration of action as well as the substances and concentrations present within. Considering that postbiotics’ application against caries primarily exerts a brief local effect within the oral cavity, and that some postbiotics in some studies take longer to be effective, some postbiotics may need to be considered in future studies in conjunction with other anticaries substances to shorten the time of entry into force. Research has revealed that the anticavity impact of postbiotics is enhanced when combined with other medications. For example, utilising lipoteichoic acid from Lactobacillus plantarum alone can suppress the production of multispecies oral biofilms, and this effect is amplified when combined with traditional intracanal medicines.^54^ Furthermore, *in vitro* combination of cetylpyridinium chloride and arginine can improve anti-biofilm efficacy.^57^

#### Current status of applications

Technological advancements are streamlining postbiotics production, thereby broadening their potential applications across multiple industries and products.^69^ Besides, as public awareness of dental caries prevention and treatment increases, there is a rising demand for products aimed at caries prevention and treatment. Moreover, dentists acknowledge that solely relying on time-consuming and expensive restorations falls short in effectively managing caries; successful caries management requires ongoing patient engagement throughout their lives. Postbiotics are widely accessible and affordable, making the market potential for postbiotics in dental caries prevention and control significant. An increasing number of companies are dedicating resources to the research and development of such products, with some achieving considerable market success. Despite the popularity of postbiotic foods and beverages for gut health and weight management, a gap exists in the market for postbiotic anticaries products.^69^ Recent studies indicate that postbiotics could play a role in modulating susceptibility to dental caries. Although this research area is nascent, the outlook is positive. It is expected that continued research advances will result in the creation of comprehensive products designed to tackle the various factors contributing to susceptibility to dental caries.

#### Future applications’ directions

**Fig 5:**
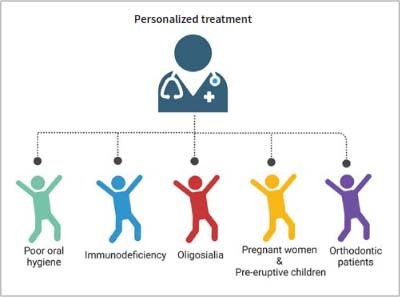
Potential application of postbiotics in personalised caries treatment. The human body suffers from caries for many reasons, which may be due to congenital dental dysplasia, insufficient salivary secretion, persistent acid production by oral cariogenic bacteria, low immunity, and so on. Postbiotics can affect multiple caries-causing factors, so in the future, specific postbiotics can be developed for different caries-causing factors to personalise the treatment of caries.

Compared to postbiotics with multiple anticaries mechanisms, single-mechanism postbiotics may have a significantly reduced anticaries effect, or might not achieve the desired outcome. For instance, certain probiotics only modulate the immune response to combat caries, a method insufficient for caries cure. Thus, it may be necessary to combine them with other anticaries agents or switch to postbiotics from other microorganisms that offer multiple anticaries mechanisms.^54,57^ Moreover, given the diverse anticaries potential of postbiotics, developing specific postbiotics aimed at various cariogenic factors could enhance personalised dental caries treatments (Fig 5). For example, patients with poor oral hygiene might benefit from postbiotics that regulate oral microbiota and promote enamel remineralisation. In cases of congenital or acquired immunodeficiency, the use of postbiotics that stimulate oral immunity may be advantageous. Orthodontic patients, at risk of early caries, could gain from postbiotics that improve remineralisation. For insufficient or poor-quality saliva, using postbiotics to enhance saliva production may be effective. For pregnant women and children before tooth eruption, postbiotics promoting tooth development and calcification are recommended. Currently, anticaries postbiotics are mainly used in toothpaste and oral rinse, but the future may bring edible or drinkable anticaries postbiotic products, simplifying caries prevention and management.

## CONCLUSIONS

Today’s consumers in the biological market are much more aware of the definition and benefits of probiotics compared to postbiotics, likely due to the nascent stage of postbiotic research and the limited availability of related products. However, given the promising prospects for anticaries postbiotics, numerous companies are already reshaping the commercial landscape. The commercialisation of postbiotics is closely linked to foundational research support. There exists a substantial body of literature on the capacity of postbiotics to mitigate caries-causing factors, yet few reviews focus on postbiotics and caries. To our knowledge, this is the inaugural review specifically addressing postbiotics and caries. Upon reviewing the literature, it is evident that some discussions of the anticaries mechanisms and related processes of postbiotics are somewhat one-sided, while others predominantly focus on or adhere to a macro-level analysis and evaluation. This review explores the anticaries mechanisms of specific postbiotic components, analyses the shortcomings and gaps in current research, and suggests future research directions in the development of anticaries postbiotics. We anticipate that this study will spur the development of additional postbiotic products to prevent and manage caries.
